# Validation of genetic variants from NGS data using deep convolutional neural networks

**DOI:** 10.1186/s12859-023-05255-7

**Published:** 2023-04-20

**Authors:** Marc Vaisband, Maria Schubert, Franz Josef Gassner, Roland Geisberger, Richard Greil, Nadja Zaborsky, Jan Hasenauer

**Affiliations:** 1 Department of Internal Medicine III with Haematology, Medical Oncology, Haemostaseology, Infectiology and Rheumatology, Oncologic Center; Salzburg Cancer Research Institute - Laboratory for Immunological and Molecular Cancer Research (SCRI-LIMCR); Cancer Cluster Salzburg, Paracelsus Medical University, Salzburg, Austria; 2 Life and Medical Sciences Institute, University of Bonn, Bonn, Germany

**Keywords:** Next-generation sequencing, Machine learning, Somatic variants

## Abstract

Accurate somatic variant calling from next-generation sequencing data is one most important tasks in personalised cancer therapy. The sophistication of the available technologies is ever-increasing, yet, manual candidate refinement is still a necessary step in state-of-the-art processing pipelines. This limits reproducibility and introduces a bottleneck with respect to scalability. We demonstrate that the validation of genetic variants can be improved using a machine learning approach resting on a Convolutional Neural Network, trained using existing human annotation. In contrast to existing approaches, we introduce a way in which contextual data from sequencing tracks can be included into the automated assessment. A rigorous evaluation shows that the resulting model is robust and performs on par with trained researchers following published standard operating procedure.

## Content

In Section “Introduction”, we introduce the reader to the problem of somatic variant refinement, why it is pertinent, and summarise the state of the art. Section “Methods” serves to detail the proposed method of performing refinement using a convolutional neural network. Its implementation is discussed in Section “Implementation”. In Section “Results”, we show its evaluation on two large datasets. Finally, section 5 contains a discussion of the results of this study.

## Introduction

Over the last decades, extensive research has been conducted to unravel the molecular, cellular and immunological mechanisms involved in cancer development. Especially the extensive use of next-generation sequencing (NGS) technologies enabled a progressively more cost-effective approach for the discovery of genetic variants within the genome that are inherited (germline mutations) or acquired (somatic mutations) and thereby predispose for or contribute to cancer development. So far, a huge amount of NGS data has been systematically analysed to determine meaningful information on potential drivers of tumourigenesis, dynamics of tumour evolution and differences between tumour entities regarding average mutational burden, tumour-specific mutational signatures or genetic diversification contributing to drug resistance and disease relapse [1–4].

In personalised cancer therapy, a common task is to identify somatic mutations from paired samples of tumour and normal tissue of individual patients to select appropriate treatment options. In a typical processing pipeline for tumour-normal pairs, the sequenced reads for each sample are aligned to a reference genome, before undergoing removal of duplicate fragments and base quality score recalibration (BQSR). This process yields NGS data ready for further analysis (most commonly stored in the binary “BAM” file format), and the normal (“germline”) can be compared to the tumour sample to obtain somatic mutations in a process known as variant calling.

Due to the importance of variant analysis in modern biomedical research, the field has seen the emergence of a number of variant calling algorithms. Their diversity in methodology can be observed in Table 1 (which distinguishes variant callers based on the categories proposed in [5]), and there has been very little consolidation in the field. Despite comprehensive large-scale benchmarking, no single configuration of tools has emerged as the approach of choice for all settings, with them all having respective advantages and drawbacks (see e.g. [5–7]). It is currently common practice to cross-reference predictions made by several different variant callers [7, 8].

**Table 1:** Variant calling tools

Strategy	Representatives
Heuristic thresholds with statistical testing	VarScan [9], VarScan2 [10], qSNP [11], Shimmer [12], RADIA [13], SOAPsnv [14], VarDict [15], and UVC [16]
Joint genotype analysis	SAMtools [17], SomaticSniper [18], JointSNVMix2 [19], Virmid [20], Seurat [21], FaSD-somatic [22], SNVSniffer [23], and CaVEMan [24]
Allele frequency analysis	Strelka [25], LoFreq [26], deepSNV [27], MuTect [28], EBCall [29], MuSE [30], LoLoPicker [31], and Strelka2 [32],
Haplotype-based strategy	FreeBayes [33], Platypus [34], HapMuC [35], LocHap [36], Longshot [37], and MuTect2 [38]
Machine learning	MutationSeq [39], BAYSIC [40], SomaticSeq [41], SNooPer [42], DeepVariant [43], Clairvoyante [44], NeuSomatic [45], and DNN-Boost [46]

**Fig. 1:**
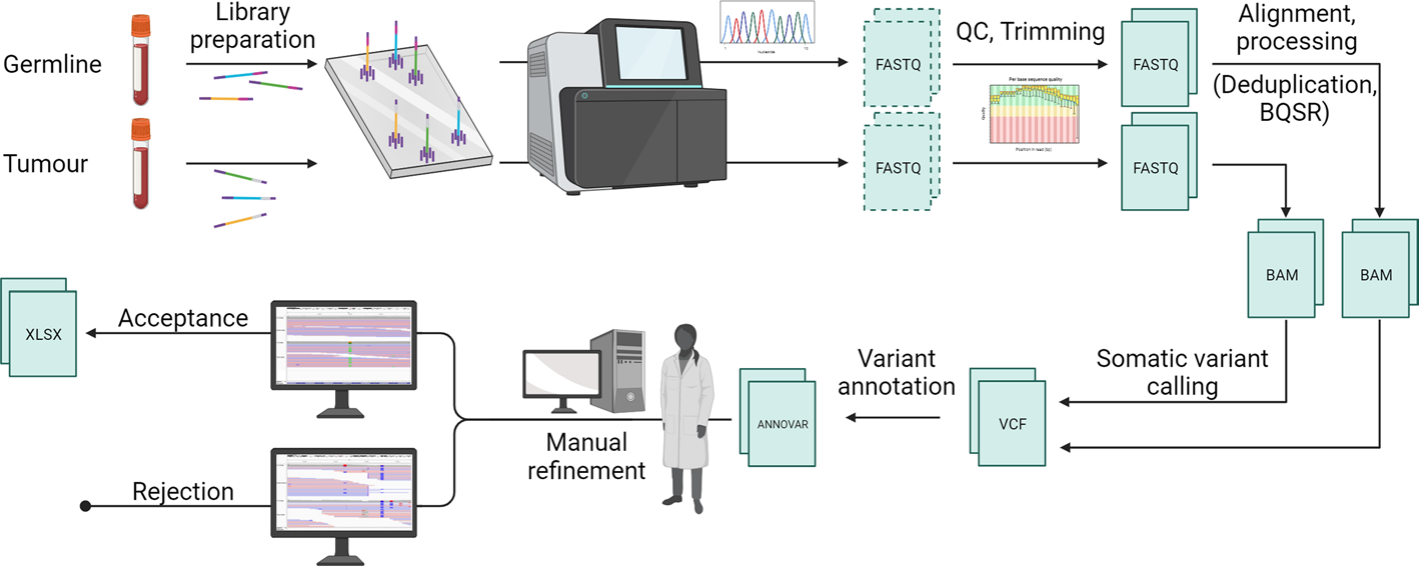
A typical processing pipeline from blood sample to somatic variant list

The brief structure of a variant calling workflow is as follows (for an overview, see Fig. 1): The genomic samples from both germline and tumour are converted into a library of fragments, which can then be sequenced yielding raw reads collected in the FASTQ format. The resulting files undergo a number of processing steps, including the trimming of low-quality sections from reads, base quality score recalibration (BQSR), and deduplication, followed by alignment against a reference genome and storage in the binary alignment map (BAM) format.

From this format, in turn, the sequencing results can be compared by a variant calling tool to generate a list of candidate variants, typically stored in a variant call format (VCF) file. Any modern variant calling workflow, however, must still incorporate a stage where these candidate variants are manually refined following published standard operating procedure by trained researchers [47–49].

Besides being immensely time-consuming, this is intrinsically at odds with the principles of reproducible research, as there is by necessity a grey area where different researchers may come to different conclusions when examining a candidate variant, despite following the same guidelines (Barnell et al. [48] give a figure of 94.1% for the accuracy of reviewers following the proposed procedure). Coupled with the fact that different pipelines may yield very different results, this points to a great need for uniformisation, something which has been clear since the advent of NGS [50]. Very recent studies, too, point to the immense need for automation in the field, as the huge diversity in variants makes manual curation infeasible [51].

One of the reasons for the need for manual refinement is that available pipelines still struggle with sequencing artefacts. The vast improvement in sequencing throughput capacity and cost-efficiency brought about by NGS has had a principal trade-off in reliability and accuracy (see e.g. [17, 52]). This bargain has been whole-heartedly embraced by the majority of the research community; it means, however, that sequencing artefacts are a common occurrence and must be accounted for when designing analysis pipelines.

**Fig. 2:**
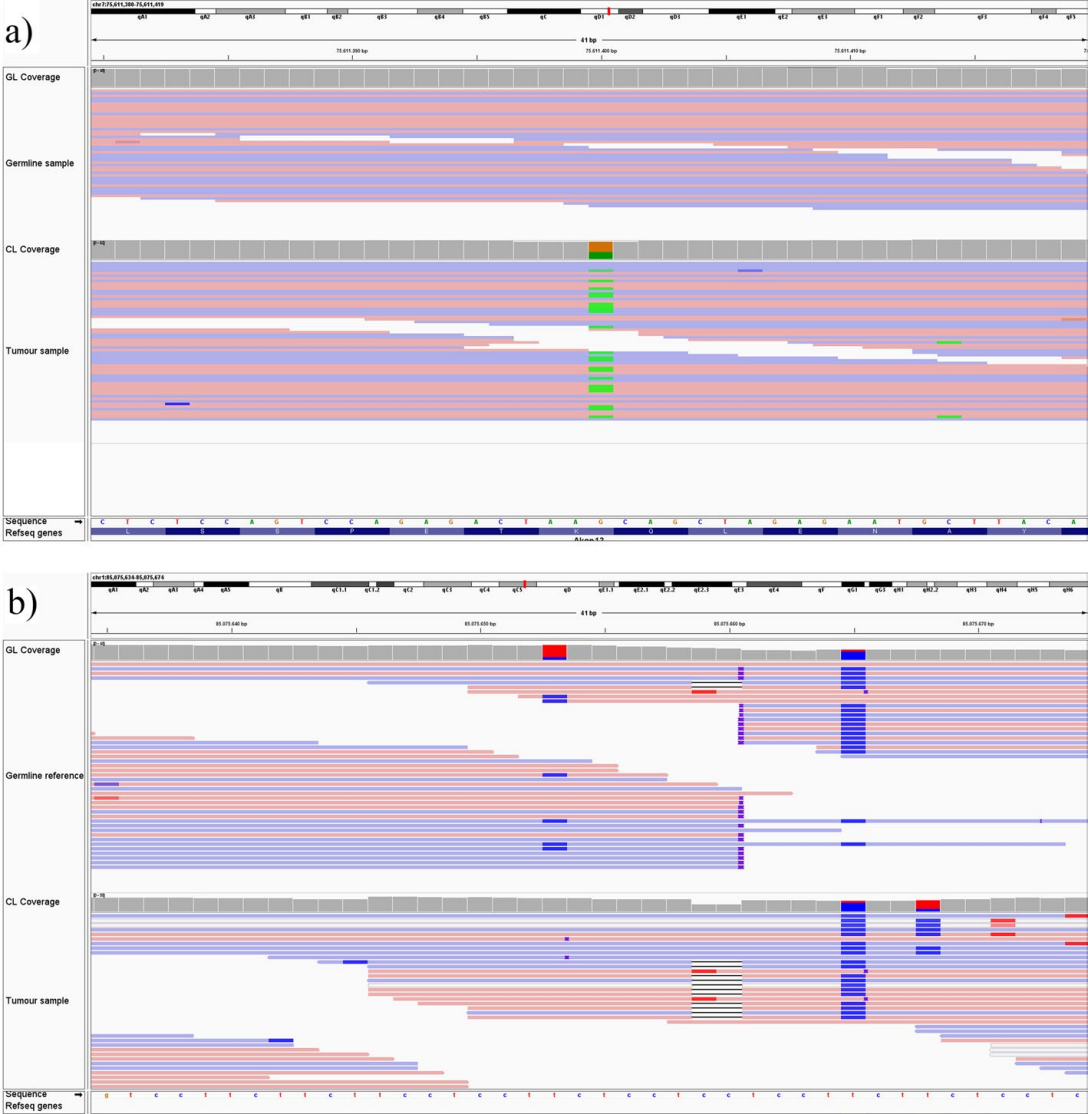
Examples of sequencing artefacts. Screen captures from the Integrative Genomics Viewer (IGV) [53]; in each case the germline track is shown in the upper half, and the tumour track in the lower. Shown are typical examples of (**a**), a genuine mutation, and (**b**), a sequencing artefact flagged as a candidate variant. The colour of the reads indicates their direction, unless they disagree with the reference genome, in which case the disagreeing base is coloured in. The artefact can be recognised by the low complexity of the DNA sequence and the subsequently misaligned abruptly ending reads, as well as general sequencing “noise”

To accurately assess whether a candidate variant is genuine or falsely called as the result of an artefact, it has to be carefully examined in specialised software. Most commonly, the Integrative Genomics Viewer (IGV) [53] is used (Fig. 2 provides a typical example of an artefact inspected using this tool). Researchers have to take into account both the quantity and quality of variant evidence, while considering factors such as low sequencing coverage, misaligned reads, strand bias, low base and alignment qualities and sequencing errors around the locus, which are especially common in regions of low complexity [48].

Artefacts can have many different sources and errors can be introduced at any step of the sequencing process, including library preparation. They may arise from sequencing during cluster amplification, cycle sequencing or image analysis. During library preparation, factors such as differential amplification, polymerase errors or the method and chemistry of DNA fragmentation can contribute to the generation of artefacts [54–58].

If the library preparation is done at the same time for both germline and tumour, this poses no problem, as the incorrectly called base will be present in both samples and no artefactual candidate variant is generated. If, however, the library preparations of germline and tumour sample differ, this can result in calls which appear highly credible at first glance. They can only be identified by consulting other, otherwise unrelated, samples which have been sequenced using the same library preparation. Coming across these artefacts is quotidian in research practice and manual variant refinement (see Additional file 1: Figures S1 and S2 in appendix B for two examples), but was until now, to our knowledge, not implemented in any refinement tool. To exclude this error source entirely, germline and tumour samples would always have to be sequenced with the same library preparation. In longitudinal studies or transplantation experiments in particular, this would necessitate a new library preparation and full re-analysis of germline samples for each transplantation. In most applications, this is infeasible and prohibitively costly.

As evidenced by the multitude of error sources, the standardisation of variant refinement is one of the most important challenges in the field today. The field of tools to automate this task remains very narrow to this day, however, especially in contrast with the large number of available variant callers. For germline variants, Li et al. [59] have proposed a Random Forest Classifier model operating on hand-crafted summary statistics. In the setting of detecting structural variants, i.e. large genomic alterations encompassing 100 or more base pairs, Liu et al. [60] have presented an approach resting on deep learning and thus data-driven features, where read alignments are converted to images for classification.

For somatic variants, however, no computational state-of-the-art exists at the time of writing. The idea to automate variant refinement using machine learning was pioneered in a seminal publication by Ainscough et al. [61]. In it, the authors presented the application of a one-layer perceptron network, as well as a Random Forest Classifier, to hand-crafted summary statistics extracted from sequencing data. This approach made it possible to incorporate a high degree of expert knowledge into their method. However, it also limits their models’ applicability to new data, as the input features included elements specific to their study (notably, one of the most significant features was the identity of the human reviewer annotating a variant). Wardell et al. [62] have published software to facilitate the application of user-defined filtering criteria to candidate variants, providing a valuable tool to streamline the manual review process. The necessity itself for a manual refinement, however, has stayed unchanged so far (see also Table 2).

In this study, we propose a deep learning method, which we will for brevity refer to as deepCNNvalid, for the automatic refinement of somatic variant lists provided by variant calls, which relies on learned features and can incorporate additional context sequencing tracks. It employs convolutional neural networks, a class of artificial neural networks that rely on “learning” (i.e. optimising with respect to a loss function) convolutional filters, which are applied across spatially arranged data. This makes them excel at tasks which are informed by local structure. They were brought into the spotlight of machine learning research by the immensely influential work of Krizhevsky, Sutskever and Hinton in [63] in the field of image classification. Since this renewal of interest, they have been used to great success in many fields, among them image processing, biomedical applications, and intersections between the two (see for example [64–68] and the citations contained therein). We demonstrate that the proposed method achieves a high degree of accuracy on different datasets.

**Table 2:** Variant refinement approaches

Name	Target setting	Features	Underlying method	Reusable model	Includes sequencing context information
Ainscough et al. [61]	Somatic	Hand-crafted, summary statistics	Random forest, one-layer perceptron	No	No
FiNGS [62]	Somatic	Hand-crafted	User-defined filtering criteria	N/A	No
deepCNNvalid (proposed method)	Somatic	Learned	CNN on reads	Yes	Yes
DeepSVFilter [60]	Structural	Learned	CNN on images	Yes	No
ForestQC [59]	Germline	Hand-crafted, summary statistics	Random Forest	Yes	No

## Methods

In this section, we introduce the deep learning method for variant refinement. Its inputs are BAM files obtained by sequence alignment, VCF files obtained by variant calling, and metainformation (e.g. on library preparation). The output is a list of refined variants from which sequencing artefacts have been removed. Below, we discuss the considered dataset and the evaluation of the method.

### Input data representation

The application of deep learning methods for variant refinement requires a numerical representation of the sequencing data. In this study, we encode the input data as a three-dimensional tensor, with the dimensions corresponding to

- Position of the base on the reference genome,
- Index of the read, and
- Base-wise information (nucleotide type, quality scores, and read direction).

We consider a fixed symmetrical window of $d_\text {window}$ bases around the potential variants listed in the VCF files provided by variant callers and a maximum of $d_\text {reads}$ reads. Base-wise information are the nucleotide type, base-wise quality, read alignment quality, as well as a binary flag showing whether a read was reversed. For the nucleotide type, a one-hot encoding is employed, resulting in four entries (one for each nucleotide), as is typically the case in Deep Learning with sequencing data [69–71]. Overall, we have seven entries per base—but this can be extended by further input features if desired. Whenever a read did not cover a position in the base window, or there were fewer than $d_\text {reads}$ reads available, the corresponding positions in the tensor were zero-padded. Overall, this process yields tensors in ${\mathbb {R}}^{d_\text {window} \times d_\text {reads} \times 7}$.

To assess the presence or absence of a variant at a specific locus, we apply this encoding scheme to the sequencing data from the tumour and the normal tracks, as well as randomly chosen sequencing tracks with no biological relation to the variant (i.e. a different transplantation line), but the same library preparation. The additional tracks are used to provide context information, e.g. on library preparation, etc.

**Fig. 3:**
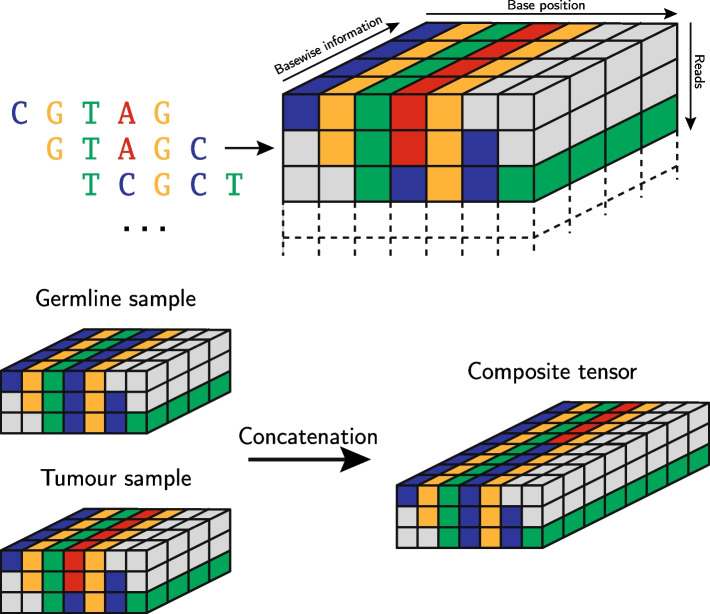
Process for obtaining composite data points from several sequencing tracks. Additional context tracks are added by the same principle

The tensors representing the information of the different sequencing tracks are concatenated along the third (i.e. “depth”) dimension. The resulting structure can be thought of as similar to the visualization researchers would see when inspecting a candidate with a genome viewer with different sequencing tracks aligned alongside each other, except that our data encoding forms a three-dimensional tensor where identical positions in the genome are aligned, cf. Figure 3.

### Model topology and training

For the variant refinement, we employed a feedforward neural network incorporating convolutional layers. This type of topology has proven valuable for various sequence analysis tasks [72]. We evaluated different structures and observed a high degree of robustness of the results. A brief discussion of different evaluated architecture features is provided in appendix D. For the subsequently presented results, the precise structure is as follows:

- Three convolutional blocks with filters of size $3 \times 3$ each (and ReLU activation). Using 32, 16 and 32 filters, respectively, each followed by Max-Pooling with the same filter size.
- One layer of $1 \times 1$ convolutions with 32 filters and ReLU activation
- A batch normalization layer with a momentum parameter of 0.8 followed by flattening
- A dense layer with 10 neurons and ReLU activation, and dropout of probability 0.2 applied on the out-edges for training
- A classification layer with two neurons and softmax activation

**Fig. 4:**
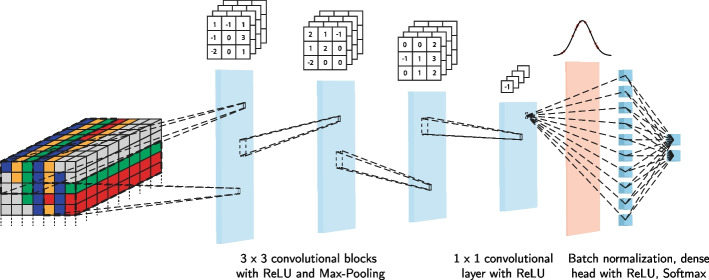
Architecture of neural network for classification. Input data runs through blocks of 3$\times$3 convolutions with ReLU activation and max-pooling, before weighted averages over the filters are taken using $1\times 1$ convolutions with ReLU. The results are flattened, normalised and passed to a dense classification head, where dropout is applied for regularisation

A visual summary is provided in Fig. 4.

Notable features of the selected architecture are the relatively small numbers of filters, and the lack of a large dense classification head which has been replaced by a $1\times 1$ convolutional layer, “forcing” complexity into the filter structure. The overall number of trainable parameters, 27, 312, is low by neural network standards, which helps avoid excessive overfitting.

For training, we chose the binary cross-entropy loss function, as is most frequently done in binary classification. For further regularization, the training labels were subjected to label smoothing with smoothing parameter 0.1, meaning that the categorical (1, 0) and (0, 1) labels were converted into (0.95, 0.05) and (0.05, 0.95) respectively. Finally, training was done with the Adam optimiser [73].

### Datasets

For the evaluation of the method we considered different datasets, focusing exclusively on candidates for single nucleotide variants (SNVs). The first dataset, generated in-house, contains 2085 candidate variants and is used for training and cross-validation. The second encompasses 1652 candidate variants and was obtained from data published in [74]. Only in-house data was used for training, with the entire out-of-facility dataset reserved for validation.

*In-facility* The in-house sequencing data came from blood samples from mice maintained at the mouse facilities of the Paris Lodron University Salzburg. To generate a first data set, we performed WES sequencing for murine samples of TCL1 and TCL1-AIDKO primary and transplanted tumours generated with the Agilent SureSelect XT Mouse All Exon Kit [75, 76]. This experimental system is a well-established widely for studying chronic lymphocytic leukemia (CLL) [77] and provided us with a highly controlled data set. The dataset includes 11 germline (GL) and 40 CLL samples (11 primary and 29 serially transplanted), based on which 40 comparisons were performed using VarScan2 [10], in order to identify somatic mutations occurring between germline and primary tumour, or transplanted tumour sample. WES data are accessible on Sequence Read Archive, NCBI, NIH (BioProject: PRJNA475208 [75], BioProject: PRJNA725403 [76] and BioProject: PRJNA789482. The variant candidates provided by VarScan2 were evaluated by three experienced researchers following published Standard Operating Procedure [48] to provide a ground-truth.

*Out-of-facility* As a second data set, the results of a mutational analysis by Kotani et al. investigating WES data from murine progenitor cells transduced with human MLL/AF9 and differentiated into bone marrow cells as a model for MLL-rearranged acute myeloid leukemia (AML) were used. Similar to the in-facility samples, primary and serially transplanted tumour samples were prepared and sequenced with the Agilent SureSelect XT Mouse All Exon V2 Kit [74]. The dataset encompasses 42 paired GL and AML samples (8 primary and 34 transplanted). Candidate variants were generated and manually annotated while cross-referencing with the list of mutations published with the data.

Details on the exact bioinformatics processing pipelines for both datasets are given in Additional file 1: Tables S1 and S2 in appendix A.

### Evaluation

**Fig. 5:**
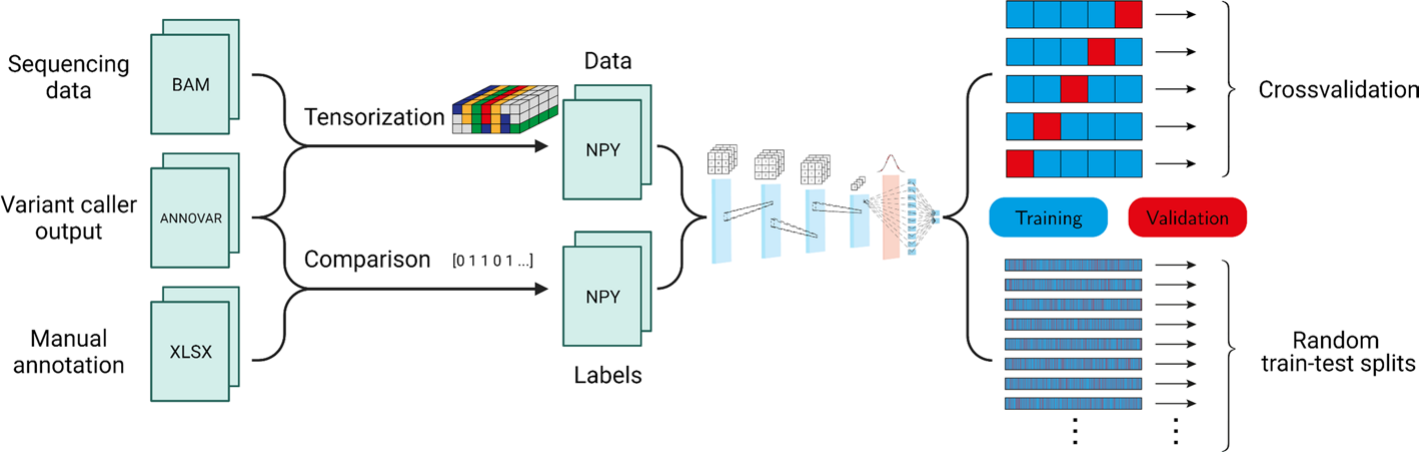
Overall model training and evaluation pipeline. The sequencing data is tensorised at candidate loci, while previous expert annotation provides labels. This dataset is then used to estimate the proposed neural network’s generalisation ability using two evaluation approaches

In the course of the overall evaluation pipeline (presented in Fig. 5), the aligned sequencing data, detected candidate variants (functionally annotated by ANNOVAR [78]), and the existing ground truth annotation were processed to numerical form and saved in the binary NPY format native to NumPy [79]. We then employed several strategies to assess the proposed method’s generalisation performance. On the in-house dataset, we used stratified 5-fold cross-validation, as well as repeated random stratified train-test splits. Additionally, the out-of-facility dataset was used as a hold-out validation set.

To contextualise the model’s performance, we compared it with the results of rule-based filtering using FiNGS [62], using both the default parameter set suggested by the authors and a more permissive one for reference (the criteria sets used are shown in Additional file 1: Appendix E).

In all cases, the model’s aptitude was assessed using the standard metrics for binary classification tasks: accuracy, recall (also known as sensitivity), precision, and F1 score. If we let *TP*, *FP*, *TN*, *FN* denote true positives, false positives, true negatives, and false negatives, respectively, we define

$$\begin{aligned} \text {Accuracy}&= \frac{TP + TN}{TP + FP + TN + FN}\\ \text {Recall}&= \frac{TP}{TP + FN} \\ \text {Precision}&= \frac{TP}{TP + FP} \\ \text {F1}&= 2 \cdot \frac{\text {Precision} \cdot \text {Recall}}{\text {Precision} + \text {Recall}} \end{aligned}$$

To gain further insights, we examined whether its accuracy on subcategories of the dataset differed significantly from the overall accuracy. In particular, we investigated possible differences in performance by reference base, alternative base, reference-alternative-pair, mutation class, and variant allele frequency (VAF). In each case, we used a binomial test to assess whether the null hypothesis that all results on subsets of the data have been obtained using the same accuracy can be rejected. The results were corrected for multiple testing using the Bonferroni method.

## Implementation

We performed the mutational analysis using the pipeline described by Schubert et al. [76]. Somatic variant calling using VarScan2 was performed using relatively permissive parameters, i.e. min-coverage-normal 5, min-coverage-tumour 5, min-var-freq 0.05, somatic-p-value 0.05 and strand-filter 1, and filtering of high confidence calls was performed according to Basic Protocol 2 published by Koboldt et al. [80], to reduce the false negative rate. The precise setting of VarScan2 dependent on the dataset, using internal standards of the clinical groups in Salzburg for the in-facility dataset and published parameters for the out-of-facility dataset (see Additional file 1: Table S2 in Appendix A). The resulting variant lists were filtered for the following mutation classes (coming from ANNOVAR analysis “Func.RefGene”): “exonic”, “ncRNA_exonic”, “splicing”, “UTR3”, “UTR5”, “UTR5;UTR3”, “downstream”, “upstream”, “upstream;downstream”].

We implemented the proposed variant list refinement method using the Keras [81] interface to the TensorFlow [82] framework in Python. For training, we chose the canonical binary cross-entropy loss function, and performed optimization with Adam [73] using a constant learning rate of $10^{-3}$. Optimization steps were done in batches of 256, for 50 epochs. The evaluation was done using the implementation of stratified cross-validation and train-test-splits provided by the scikit-learn [83] toolkit for Python. For the binomial test between data subsets, we chose the implementation in the statsmodels package for Python [84].

For the tensor representation of the data, we chose a window size of $d_\text {window} = 101$ bases around the examined locus. Reads were considered up to a maximum number of $d_\text {reads} = 200$ reads from each track.

To handle potential homologies distorting the reported classification performance, we base-scrambled all input data, independently uniformly at random swapping A-T, C-G, neither pair, or both. This was done only within purine and pyrimidine bases, respectively, since otherwise biological information contained in the difference between transitional and transversional mutations would be lost [85]. Since the bases are represented in one-hot encoding, this transformation corresponded to permuting the relevant slices of each datapoint tensor.

The complete implementation is available on GitHub (https://github.com/marc-vaisband/deepCNNvalid).

The final version used for the study is archived at Zenodo (https://zenodo.org/record/6409366).

## Results

In the following, we illustrate and evaluate the accuracy of the proposed variant filtering approach. For this purpose, we consider the afore-described in- and out-of-facility datasets.

### Information on sequencing context improves classification efficiency

To evaluate the proposed method and to understand the dependence of its performance on different factors, we first studied the in-house dataset. For this the precise experimental setup is well known and the use of mouse data from several transplantation rounds provides us with additional controls (e.g. an the plausibility of the absence and presence of mutations). We processed the data as described in the *Implementation* section. This yielded 2085 data points (of them 703 mutations and 1382 artefacts), each corresponding to a particular tumour-normal sample pair and a position in the genome. To generate a ground truth datasets, the list of variant candidates was screened by three experienced researchers following the guidelines by Barnell et al. [48]. The consensus annotation by these researchers provided the corresponding labels, dividing the candidates into true mutations and artefacts.

In a first step, we performed stratified 5-fold cross-validation, using the scikit-learn [83] implementation, with a fixed random seed, splitting the in-house dataset into five folds. We find that the proposed method, which accounts for context, achieves a good performance as measured in validation accuracy, validation precision, validation recall and validation F1 score, averaged over the five stratified folds. To assess whether the method can be simplified by removing context information, we reran the evaluation with reduced input tensors and neural network. This revealed that context information indeed improves the validation performance, by 0.02 points compared to the context-free case. Most likely, this is due to added information on the overall quality of a sequencing run and sequencing artefacts. The fold-averaged scores are found in the following Table 3:

**Table 3:** Average validation performance in 5-fold cross-validation

Dataset	Accuracy	Precision	Recall	F1
With context tracks, average	0.968	0.953	0.954	0.953
Without context tracks, average	0.952	0.936	0.92	0.927

As a baseline comparison, the performance of rule-based filtering is shown in the following Table 4:

**Table 4:** Classification performance of rule-based filtering

	Accuracy	Precision	Recall	F1
Default criteria	0.899	0.986	0.711	0.826
Permissive criteria	0.941	0.939	0.882	0.910

As the training time of the model is moderate (408 s on a node with 56 CPUs and no GPU acceleration), we assessed the reliability of the results by considering 100 random stratified train-test-splits of our data. We again observe that including context tracks improves the performance metrics, by 0.01 to 0.02 points. The averaged scores over all training runs are found in the following Table 5:

**Table 5:** Average validation performance across 100 random train-test-splits

Dataset	Accuracy	Precision	Recall	F1
With context tracks, average	0.958	0.934	0.945	0.937
Without context tracks, average	0.946	0.921	0.923	0.920

A visual summary of both evaluation results can be found in Fig. 7; the detailed cross-validation scores per fold are included in Additional file 1: Tables S3 and S4 in appendix C.

### Variant candidate refinement performs well even for limited training set sizes

**Fig. 6:**
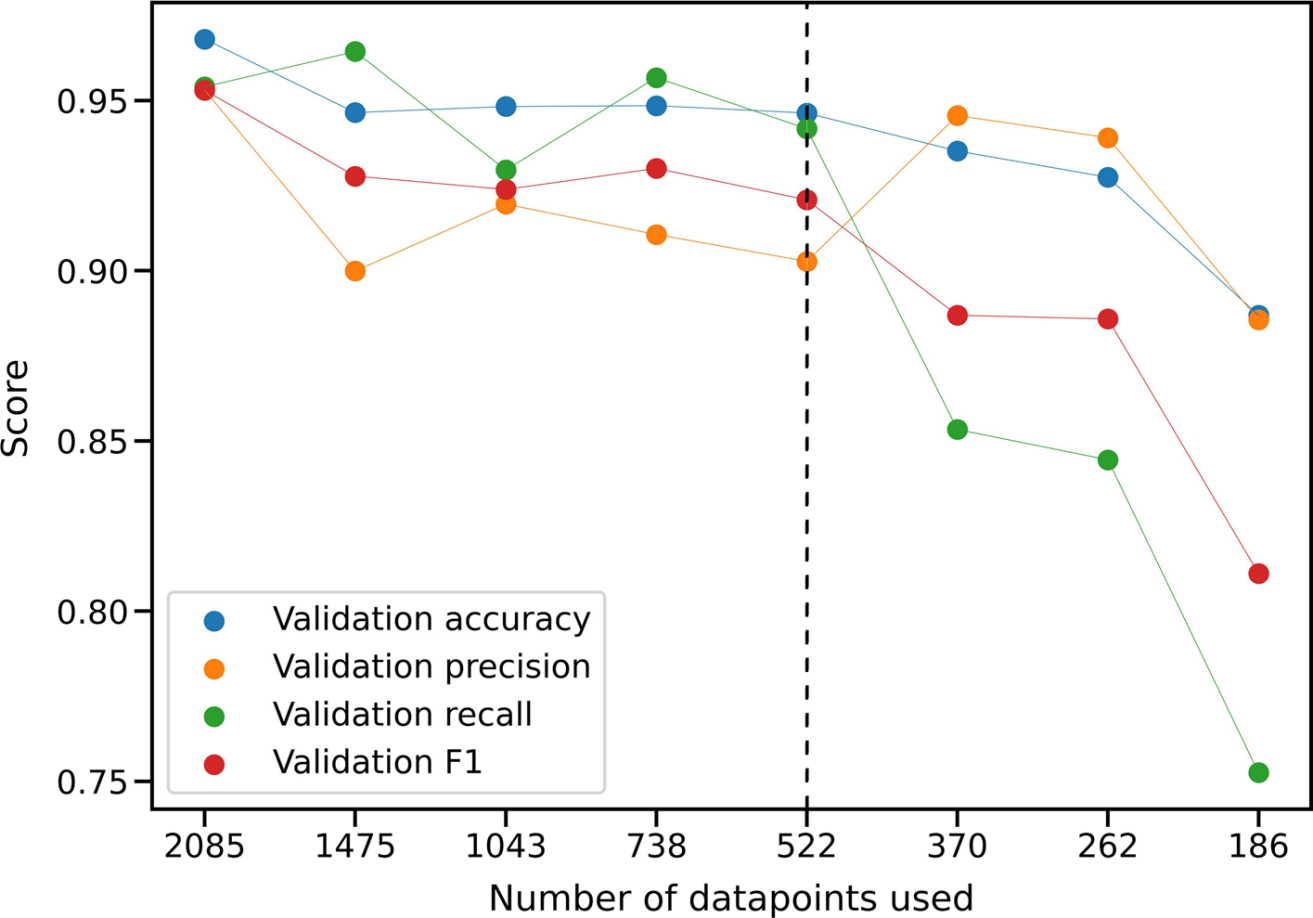
Results of data subsampling. The scores of successive cross-validation runs after repeatedly sub-sampling the data with a factor of $\sqrt{2}$. As expected, model performance worsens with fewer data points. This initially happens gradually, but becomes precipitous once the sample size falls below 500 (indicated by the dashed line)

As the proposed method performed rather well despite the relatively limited number of training examples provided by the in-house dataset, we assessed the dependence of the validation performance on sample availability. Therefore, we progressively sub-sampled the training set, repeatedly reducing its size by a factor of $\sqrt{2}$, and performed 5-fold cross-validation on each subset. We observed that as expected, overall performance declines as the number of datapoints becomes smaller. Reducing it to roughly 500 leads to worse, but still reasonable model performance; a further reduction however leads to a severe decline in classification quality (cf. Figure 6). This supports the conventional wisdom that deep learning methods often need sample sizes well into the hundreds in order to perform well.

**Fig. 7:**
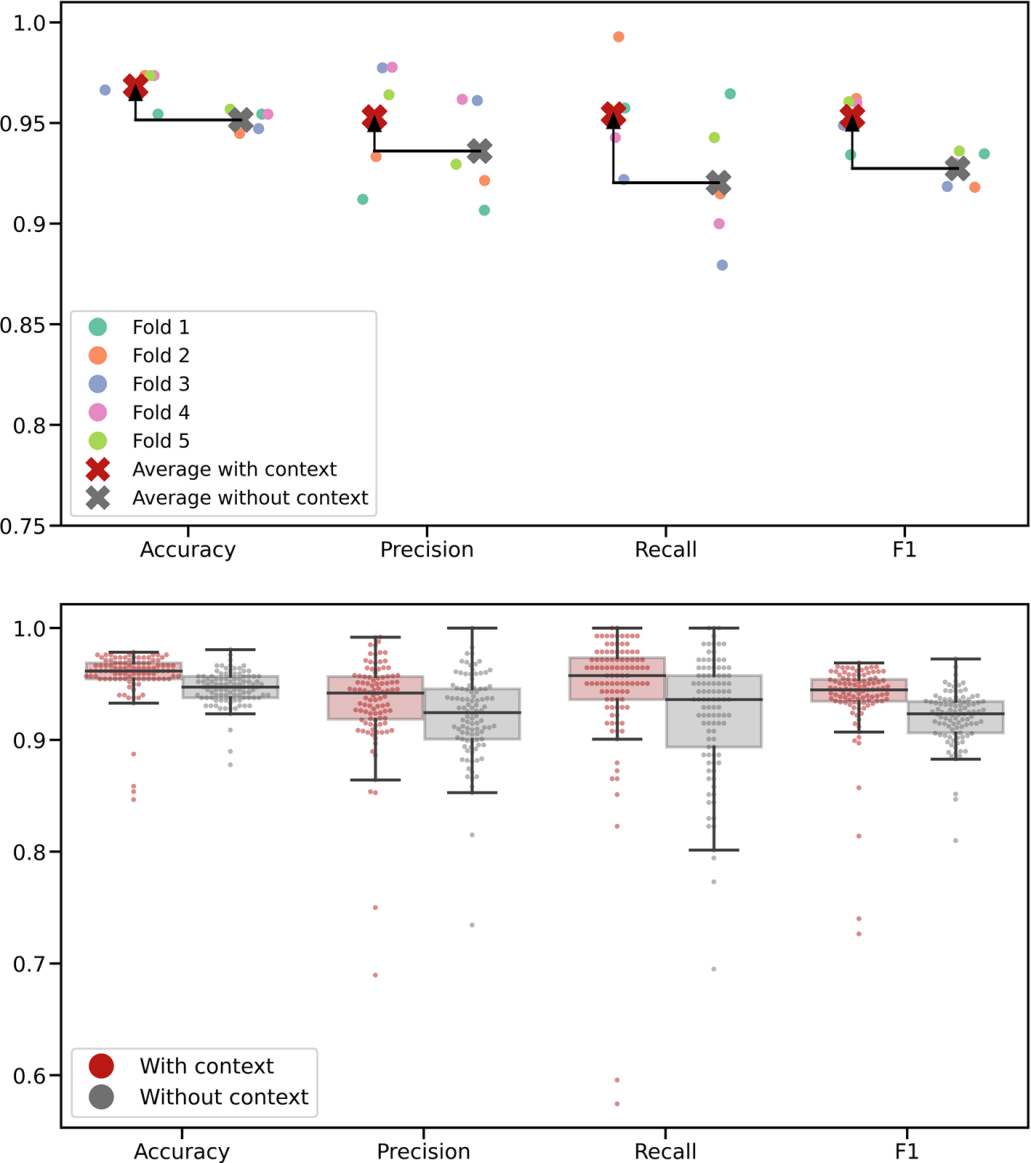
Results of model evaluation. Modelling with context tracks shown in red; without in grey. In the top figure, the results of 5-fold cross-validation are shown, with arrows to indicate the improvement achieved by including context. In the bottom figure, the results of $2\times 100$ optimization runs with random train-test splits are presented

### Classification performance does not depend on bases or mutation class

For the potential use of the proposed method in clinical practice, it is crucial to gain a precise understanding of the model’s behaviour with respect to different kinds of candidate variants. In particular unbiasedness is an important feature. To analyse this, we subdivided the in-house data based on reference base, alternative base, and mutation category. Using the validation predictions from the cross-validation, we investigated whether the validation accuracy on any one group based on these distinctions differed significantly from the overall accuracy, using a binomial test. No significant differences were found. This suggests that the model performs equally well in all cases - irrespective of the reference and alternative bases, as well as mutation category.

### The model generalises to independent out-of-facility data

As the model perform well on the in-facility data, we performed the arguably most rigorous, assessment of generalisation ability for any machine learning model is of course its application, and tested it on data which was generated completely independently of the data used for training or even in-facility validation. For this purpose, we utilised the data published in [74] and processed as outlined in the Implementation section.

We trained a model on all 2085 of our in-facility data points, and evaluated it on the 1652 candidate variants from the out-of-facility data.

The final evaluation of the out-of-facility dataset for independent validation yielded an accuracy of 0.949, a precision of 0.948, a recall of 0.995, and an F1 score of 0.971. These levels are similar to the in-facility data used for training, showing that the method generalizes to different sequencing methods and experts.

## Discussion

In this manuscript, we considered the identification of somatic variants from NGS data, which is one of the key challenges in modern cancer research. It is aided by ever-improving sequencing and variant calling technology, but currently still dependent on manual candidate refinement, which presents a huge opportunity for improvement in terms of scalability and reproducibility. Here, we demonstrate that Convolutional Neural Networks can provide accurate classifiers and proposed a concrete network topology.

Our results demonstrate that the proposed method is indeed well-suited for the task. It features low validation error rates in cross-validation and generalises equally well to independent data from a completely different facility. The achieved performance is in both cases on par with specifically trained human researchers following standard operating procedure, and achieves a better refinement than rule-based filtering. Moreover, our analysis shows that the inclusion of additional sequencing tracks for added context improves classification results by a considerable margin - while the improvement in percentage points is small due to the high overall accuracies in both cases, the error rate is cut by roughly a third.

A similar approach would likely be equally successful for the refinement of germline variants, which have recently been the subject of particularly active research in cancer biology ([86, 87]). Here, too, the recognition of technical artefacts presents a formidable challenge [88]. The same methodology as presented in this work would allow a sequenced germline sample to be tensorised and evaluated by a neural network. In particular, it should be noted that the idea of including additional context tracks to handle library-specific artefacts translates analogously to this case, so that sequencing data of unrelated samples with the same library preparation would be added along the depth dimension.

The presented results demonstrate that the suggested method is well-suited for the problem and provides the functionality of reproducing results; this does not mean, however, that the concrete model we trained should be immediately applied in other settings. Instead, further research is urgently needed on how to proceed towards a unified automated system for variant refinement which can be reliably used by researchers across different facilities and disciplines.

The pipeline presented in this manuscript was evaluated only on data from leukaemia samples, for which we had access to the necessary information, such as ground truth about sequencing artefacts, library preparation etc. As such, a confirmation of our results on different cancer entities would be highly desirable. However, this lies outside the scope of this manuscript, as the necessary metadata outlined above are typically missing from public datasets.

One important factor is that sequencing artefacts can be strongly affected by sample preparation, which differs between leukaemias and solid tumours. It is well-known that formalin fixation can cause a specific class of artefacts to arise [?,?,?], so that we firmly believe that it is an imperative to establish community standards for providing more comprehensive information about published sequencing data. This would greatly benefit not just the application of machine learning methods to sequencing data, but also transparency and reproducibility in the field as a whole.

There are also other directions which warrant further investigation. One natural avenue for extension would be the inclusion of indels, where repetitive sequences play a large role, so that we would once again expect a convolutional approach to do well. From a biological point of view, the most relevant follow-up would be a thorough investigation of the possible causes for library-specific sequencing artefacts; while they are routinely observed in practice, the authors are not aware of any comprehensive analysis of the underlying mechanisms.

To conclude, we have presented a method which can contribute to the standardisation of somatic variant calling and will benefit the research community by improving efficiency, reproducibility and interoperability. We hope that given time and data availability, tools such as this may become widely accepted in everyday research and clinical practice.

## Supplementary Information

**Additional file 1.** Appendix.

## Data Availability

All code used in this publication is available at GitHub (https://github.com/marc-vaisband/deepCNNvalid), with the final version archived at Zenodo (https://zenodo.org/record/6409366). The used WES data is available at SRA PRJNA475208 (https://www.ncbi.nlm.nih.gov/bioproject/PRJNA475208/), PRJNA725403 (https://www.ncbi.nlm.nih.gov/bioproject/PRJNA725403/) and PRJNA789482 (https://dataview.ncbi.nlm.nih.gov/object/PRJNA789482?reviewer=c8gi3p91j8s6ggq8k1k68od2ut).

## References

[1] Williams MJ, Werner B, Barnes CP, Graham TA, Sottoriva A (2016). Identification of neutral tumor evolution across cancer types. Nat Genet.

[2] Vogelstein B, Papadopoulos N, Velculescu VE, Zhou S, Diaz LA, Kinzler KW (2013). Cancer genome landscapes. Science.

[3] Alexandrov LB, Nik-Zainal S, Wedge DC, Aparicio SA, Behjati S, Biankin AV, Bignell GR, Bolli N, Borg A, Børresen-Dale A-L (2013). Signatures of mutational processes in human cancer. Nature.

[4] Alexandrov LB, Kim J, Haradhvala NJ, Huang MN, Ng AWT, Wu Y, Boot A, Covington KR, Gordenin DA, Bergstrom EN (2020). The repertoire of mutational signatures in human cancer. Nature.

[5] Xu C (2018). A review of somatic single nucleotide variant calling algorithms for next-generation sequencing data. Comput Struct Biotechnol J.

[6] Bartha Á, Győrffy B (2019). Comprehensive outline of whole exome sequencing data analysis tools available in clinical oncology. Cancers.

[7] Kumaran M, Subramanian U, Devarajan B (2019). Performance assessment of variant calling pipelines using human whole exome sequencing and simulated data. BMC Bioinform.

[8] Koboldt DC (2020). Best practices for variant calling in clinical sequencing. Genome Med.

[9] Koboldt DC, Chen K, Wylie T, Larson DE, McLellan MD, Mardis ER, Weinstock GM, Wilson RK, Ding L (2009). VarScan: variant detection in massively parallel sequencing of individual and pooled samples. Bioinformatics.

[10] Koboldt DC, Zhang Q, Larson DE, Shen D, McLellan MD, Lin L, Miller CA, Mardis ER, Ding L, Wilson RK (2012). VarScan 2: somatic mutation and copy number alteration discovery in cancer by exome sequencing. Genome Res.

[11] Kassahn KS, Holmes O, Nones K, Patch A-M, Miller DK, Christ AN, Harliwong I, Bruxner TJ, Xu Q, Anderson M (2013). Somatic point mutation calling in low cellularity tumors. PLoS ONE.

[12] Hansen NF, Gartner JJ, Mei L, Samuels Y, Mullikin JC (2013). Shimmer: detection of genetic alterations in tumors using next-generation sequence data. Bioinformatics.

[13] Radenbaugh AJ, Ma S, Ewing A, Stuart JM, Collisson EA, Zhu J, Haussler D (2014). RADIA: RNA and DNA integrated analysis for somatic mutation detection. PLoS ONE.

[14] Shi Y. SOAPsnv: An integrated tool for somatic single-nucleotide variants detection with or without normal tissues in cancer genome. Am Soc Clin Oncol. 2014.

[15] Lai Z, Markovets A, Ahdesmaki M, Chapman B, Hofmann O, McEwen R, Johnson J, Dougherty B, Barrett JC, Dry JR (2016). VarDict: a novel and versatile variant caller for next-generation sequencing in cancer research. Nucleic Acids Res.

[16] Zhao X, Hu A, Wang S, Wang X. Calling small variants with universality and. 2021. 10.1093/bib/bbab458 34791010

[17] Li H (2011). A statistical framework for SNP calling, mutation discovery, association mapping and population genetical parameter estimation from sequencing data. Bioinformatics.

[18] Larson DE, Harris CC, Chen K, Koboldt DC, Abbott TE, Dooling DJ, Ley TJ, Mardis ER, Wilson RK, Ding L (2012). SomaticSniper: identification of somatic point mutations in whole genome sequencing data. Bioinformatics.

[19] Roth A, Ding J, Morin R, Crisan A, Ha G, Giuliany R, Bashashati A, Hirst M, Turashvili G, Oloumi A (2012). JointSNVMix: a probabilistic model for accurate detection of somatic mutations in normal/tumour paired next-generation sequencing data. Bioinformatics.

[20] Kim S, Jeong K, Bhutani K, Lee JH, Patel A, Scott E, Nam H, Lee H, Gleeson JG, Bafna V (2013). Virmid: accurate detection of somatic mutations with sample impurity inference. Genome Biol.

[21] Christoforides A, Carpten JD, Weiss GJ, Demeure MJ, Von Hoff DD, Craig DW (2013). Identification of somatic mutations in cancer through Bayesian-based analysis of sequenced genome pairs. BMC Genom.

[22] Wang W, Wang P, Xu F, Luo R, Wong MP, Lam T-W, Wang J (2014). FaSD-somatic: a fast and accurate somatic SNV detection algorithm for cancer genome sequencing data. Bioinformatics.

[23] Liu Y, Loewer M, Aluru S, Schmidt B (2016). SNVSniffer: an integrated caller for germline and somatic single-nucleotide and indel mutations. BMC Syst Biol.

[24] Jones D, Raine KM, Davies H, Tarpey PS, Butler AP, Teague JW, Nik-Zainal S, Campbell PJ (2016). cgpCaVEManWrapper: simple execution of CaVEMan in order to detect somatic single nucleotide variants in NGS data. Curr Protoc Bioinformatics.

[25] Saunders CT, Wong WS, Swamy S, Becq J, Murray LJ, Cheetham RK (2012). Strelka: accurate somatic small-variant calling from sequenced tumor-normal sample pairs. Bioinformatics.

[26] Wilm A, Aw PPK, Bertrand D, Yeo GHT, Ong SH, Wong CH, Khor CC, Petric R, Hibberd ML, Nagarajan N (2012). LoFreq: a sequence-quality aware, ultra-sensitive variant caller for uncovering cell-population heterogeneity from high-throughput sequencing datasets. Nucleic Acids Res.

[27] Gerstung M, Beisel C, Rechsteiner M, Wild P, Schraml P, Moch H, Beerenwinkel N (2012). Reliable detection of subclonal single-nucleotide variants in tumour cell populations. Nat Commun.

[28] Cibulskis K, Lawrence MS, Carter SL, Sivachenko A, Jaffe D, Sougnez C, Gabriel S, Meyerson M, Lander ES, Getz G (2013). Sensitive detection of somatic point mutations in impure and heterogeneous cancer samples. Nat Biotechnol.

[29] Shiraishi Y, Sato Y, Chiba K, Okuno Y, Nagata Y, Yoshida K, Shiba N, Hayashi Y, Kume H, Homma Y (2013). An empirical Bayesian framework for somatic mutation detection from cancer genome sequencing data. Nucleic Acids Res.

[30] Fan Y, Xi L, Hughes DS, Zhang J, Zhang J, Futreal PA, Wheeler DA, Wang W (2016). MuSE: accounting for tumor heterogeneity using a sample-specific error model improves sensitivity and specificity in mutation calling from sequencing data. Genome Biol.

[31] Carrot-Zhang J, Majewski J (2017). LoLoPicker: detecting low allelic-fraction variants from low-quality cancer samples. Oncotarget.

[32] Kim S, Scheffler K, Halpern AL, Bekritsky MA, Noh E, Källberg M, Chen X, Kim Y, Beyter D, Krusche P (2018). Strelka2: fast and accurate calling of germline and somatic variants. Nat Methods.

[33] Garrison E, Marth G. Haplotype-based variant detection from short-read sequencing. 2012. arXiv preprint arXiv:1207.3907

[34] Rimmer A, Phan H, Mathieson I, Iqbal Z, Twigg SR, Wilkie AO, McVean G, Lunter G (2014). Integrating mapping-, assembly-and haplotype-based approaches for calling variants in clinical sequencing applications. Nat Genet.

[35] Usuyama N, Shiraishi Y, Sato Y, Kume H, Homma Y, Ogawa S, Miyano S, Imoto S (2014). HapMuC: somatic mutation calling using heterozygous germ line variants near candidate mutations. Bioinformatics.

[36] Sengupta S, Gulukota K, Zhu Y, Ober C, Naughton K, Wentworth-Sheilds W, Ji Y (2016). Ultra-fast local-haplotype variant calling using paired-end DNA-sequencing data reveals somatic mosaicism in tumor and normal blood samples. Nucleic Acids Res.

[37] Edge P, Bansal V (2019). Longshot enables accurate variant calling in diploid genomes from single-molecule long read sequencing. Nat Commun.

[38] Benjamin D, Sato T, Cibulskis K, Getz G, Stewart C, Lichtenstein L. Calling somatic snvs and indels with mutect2. BioRxiv, 861054. 2019.

[39] Ding J, Bashashati A, Roth A, Oloumi A, Tse K, Zeng T, Haffari G, Hirst M, Marra MA, Condon A (2012). Feature-based classifiers for somatic mutation detection in tumour-normal paired sequencing data. Bioinformatics.

[40] Cantarel BL, Weaver D, McNeill N, Zhang J, Mackey AJ, Reese J (2014). Baysic: a Bayesian method for combining sets of genome variants with improved specificity and sensitivity. BMC Bioinform.

[41] Fang LT, Afshar PT, Chhibber A, Mohiyuddin M, Fan Y, Mu JC, Gibeling G, Barr S, Asadi NB, Gerstein MB (2015). An ensemble approach to accurately detect somatic mutations using SomaticSeq. Genome Biol.

[42] Spinella J-F, Mehanna P, Vidal R, Saillour V, Cassart P, Richer C, Ouimet M, Healy J, Sinnett D (2016). SNooPer: a machine learning-based method for somatic variant identification from low-pass next-generation sequencing. BMC Genom.

[43] Poplin R, Chang P-C, Alexander D, Schwartz S, Colthurst T, Ku A, Newburger D, Dijamco J, Nguyen N, Afshar PT (2018). A universal SNP and small-indel variant caller using deep neural networks. Nat Biotechnol.

[44] Luo R, Sedlazeck FJ, Lam T-W, Schatz MC. Clairvoyante: a multi-task convolutional deep neural network for variant calling in single molecule sequencing. bioRxiv, 310458. 2018. 10.1038/s41467-019-09025-z PMC6397153 30824707

[45] Sahraeian SME, Liu R, Lau B, Podesta K, Mohiyuddin M, Lam HY (2019). Deep convolutional neural networks for accurate somatic mutation detection. Nat Commun.

[46] Maruf FA, Pratama R, Song G (2021). DNN-Boost: Somatic mutation identification of tumor-only whole-exome sequencing data using deep neural network and XGBoost. J Bioinform Comput Biol.

[47] Roy S, Coldren C, Karunamurthy A, Kip NS, Klee EW, Lincoln SE, Leon A, Pullambhatla M, Temple-Smolkin RL, Voelkerding KV (2018). Standards and guidelines for validating next-generation sequencing bioinformatics pipelines: a joint recommendation of the association for molecular pathology and the college of American pathologists. J Mol Diagn.

[48] Barnell EK, Ronning P, Campbell KM, Krysiak K, Ainscough BJ, Sheta LM, Pema SP, Schmidt AD, Richters M, Cotto KC (2019). Standard operating procedure for somatic variant refinement of sequencing data with paired tumor and normal samples. Genet Med.

[49] Cigdem SB, Yuval I (2020). Identifying disease-causing mutations in genomes of single patients by computational approaches. Hum Genet.

[50] O’Rawe J, Jiang T, Sun G, Wu Y, Wang W, Hu J, Bodily P, Tian L, Hakonarson H, Johnson WE (2013). Low concordance of multiple variant-calling pipelines: practical implications for exome and genome sequencing. Genome Med.

[51] Doig KD, Love CG, Conway T, Seleznev A, Ma D, Fellowes A, Blombery P, Fox SB (2022). Findings from precision oncology in the clinic: rare, novel variants are a significant contributor to scaling molecular diagnostics. BMC Med Genom.

[52] Shendure J, Ji H (2008). Next-generation DNA sequencing. Nat Biotechnol.

[53] Robinson JT, Thorvaldsdóttir H, Winckler W, Guttman M, Lander ES, Getz G, Mesirov JP (2011). Integrative genomics viewer. Nat Biotechnol.

[54] Schmitt MW, Kennedy SR, Salk JJ, Fox EJ, Hiatt JB, Loeb LA (2012). Detection of ultra-rare mutations by next-generation sequencing. Proc Natl Acad Sci.

[55] Kinde I, Wu J, Papadopoulos N, Kinzler KW, Vogelstein B (2011). Detection and quantification of rare mutations with massively parallel sequencing. Proc Natl Acad Sci.

[56] Park G, Park JK, Shin S-H, Jeon H-J, Kim NK, Kim YJ, Shin H-T, Lee E, Lee KH, Son D-S (2017). Characterization of background noise in capture-based targeted sequencing data. Genome Biol.

[57] Gregory T, Ngankeu A, Orwick S, Kautto EA, Woyach JA, Byrd JC, Blachly JS (2020). Characterization and mitigation of fragmentation enzyme-induced dual stranded artifacts. NAR Genom Bioinform.

[58] Tanaka N, Takahara A, Hagio T, Nishiko R, Kanayama J, Gotoh O, Mori S (2020). Sequencing artifacts derived from a library preparation method using enzymatic fragmentation. PLoS ONE.

[59] Li J, Jew B, Zhan L, Hwang S, Coppola G, Freimer NB, Sul JH (2019). ForestQC: quality control on genetic variants from next-generation sequencing data using random forest. PLoS Comput Biol.

[60] Liu Y, Huang Y, Wang G, Wang Y (2021). A deep learning approach for filtering structural variants in short read sequencing data. Brief Bioinform.

[61] Ainscough BJ, Barnell EK, Ronning P, Campbell KM, Wagner AH, Fehniger TA, Dunn GP, Uppaluri R, Govindan R, Rohan TE (2018). A deep learning approach to automate refinement of somatic variant calling from cancer sequencing data. Nat Genet.

[62] Wardell CP, Ashby C, Bauer MA (2021). FiNGS: high quality somatic mutations using filters for next generation sequencing. BMC Bioinform.

[63] Krizhevsky A, Sutskever I, Hinton GE (2012). Imagenet classification with deep convolutional neural networks. Adv Neural Inf Process Syst.

[64] Rawat W, Wang Z (2017). Deep convolutional neural networks for image classification: a comprehensive review. Neural Comput.

[65] Anwar SM, Majid M, Qayyum A, Awais M, Alnowami M, Khan MK (2018). Medical image analysis using convolutional neural networks: a review. J Med Syst.

[66] Bernal J, Kushibar K, Asfaw DS, Valverde S, Oliver A, Martí R, Lladó X (2019). Deep convolutional neural networks for brain image analysis on magnetic resonance imaging: a review. Artif Intell Med.

[67] Schwendicke F, Golla T, Dreher M, Krois J (2019). Convolutional neural networks for dental image diagnostics: a scoping review. J Dent.

[68] Tajbakhsh N, Shin JY, Gurudu SR, Hurst RT, Kendall CB, Gotway MB, Liang J (2016). Convolutional neural networks for medical image analysis: Full training or fine tuning?. IEEE Trans Med Imaging.

[69] Zhou J, Troyanskaya OG (2015). Predicting effects of noncoding variants with deep learning-based sequence model. Nat Methods.

[70] Kelley DR, Snoek J, Rinn JL (2016). Basset: learning the regulatory code of the accessible genome with deep convolutional neural networks. Genome Res.

[71] Alipanahi B, Delong A, Weirauch MT, Frey BJ (2015). Predicting the sequence specificities of DNA-and RNA-binding proteins by deep learning. Nat Biotechnol.

[72] Schmidt B, Hildebrandt A (2021). Deep learning in next-generation sequencing. Drug Discov Today.

[73] Kingma DP, Ba J. Adam: A Method for Stochastic Optimization. 2017. arXiv:1412.6980

[74] Kotani S, Yoda A, Kon A, Kataoka K, Ochi Y, Shiozawa Y, Hirsch C, Takeda J, Ueno H, Yoshizato T (2019). Molecular pathogenesis of disease progression in MLL-rearranged AML. Leukemia.

[75] Zaborsky N, Gassner FJ, Höpner JP, Schubert M, Hebenstreit D, Stark R, Asslaber D, Steiner M, Geisberger R, Greil R (2019). Exome sequencing of the TCL1 mouse model for CLL reveals genetic heterogeneity and dynamics during disease development. Leukemia.

[76] Schubert M, Gassner FJ, Huemer M, Höpner JP, Akimova E, Steiner M, Egle A, Greil R, Zaborsky N, Geisberger R (2021). Aid contributes to accelerated disease progression in the TCL1 mouse transplant model for CLL. Cancers.

[77] Bichi R, Shinton SA, Martin ES, Koval A, Calin GA, Cesari R, Russo G, Hardy RR, Croce CM (2002). Human chronic lymphocytic leukemia modeled in mouse by targeted TCL1 expression. Proc Natl Acad Sci.

[78] Wang K, Li M, Hakonarson H (2010). ANNOVAR: functional annotation of genetic variants from high-throughput sequencing data. Nucleic Acids Res.

[79] Harris CR, Millman KJ, van der Walt SJ, Gommers R, Virtanen P, Cournapeau D, Wieser E, Taylor J, Berg S, Smith NJ, Kern R, Picus M, Hoyer S, van Kerkwijk MH, Brett M, Haldane A, del Río JF, Wiebe M, Peterson P, Gérard-Marchant P, Sheppard K, Reddy T, Weckesser W, Abbasi H, Gohlke C, Oliphant TE (2020). Array programming with NumPy. Nature.

[80] Koboldt DC, Larson DE, Wilson RK (2013). Using VarScan 2 for germline variant calling and somatic mutation detection. Curr Protoc Bioinform.

[81] Chollet F, et al. Keras. https://keras.io. 2015.

[82] Abadi M, Agarwal A, Barham P, Brevdo E, Chen Z, Citro C, Corrado GS, Davis A, Dean J, Devin M, et al. Tensorflow: Large-scale machine learning on heterogeneous distributed systems. 2016. arXiv preprint arXiv:1603.04467

[83] Pedregosa F, Varoquaux G, Gramfort A, Michel V, Thirion B, Grisel O, Blondel M, Prettenhofer P, Weiss R, Dubourg V, Vanderplas J, Passos A, Cournapeau D, Brucher M, Perrot M, Duchesnay E (2011). Scikit-learn: machine learning in Python. J Mach Learn Res.

[84] Seabold S, Perktold J. Statsmodels: Econometric and statistical modeling with python. In: 9th Python in Science Conference. 2010.

[85] Rosenberg MS, Subramanian S, Kumar S (2003). Patterns of transitional mutation biases within and among mammalian genomes. Mol Biol Evol.

[86] Huang K-L, Mashl RJ, Wu Y, Ritter DI, Wang J, Oh C, Paczkowska M, Reynolds S, Wyczalkowski MA, Oak N (2018). Pathogenic germline variants in 10,389 adult cancers. Cell.

[87] Musa J, Cidre-Aranaz F, Aynaud M-M, Orth MF, Knott MM, Mirabeau O, Mazor G, Varon M, Hölting TL, Grossetête S (2019). Cooperation of cancer drivers with regulatory germline variants shapes clinical outcomes. Nat Commun.

[88] Buckley AR, Standish KA, Bhutani K, Ideker T, Lasken RS, Carter H, Harismendy O, Schork NJ (2017). Pan-cancer analysis reveals technical artifacts in TCGA germline variant calls. BMC Genom.

[89] Van Laarhoven T. L2 regularization versus batch and weight normalization. 2017. arXiv preprint arXiv:1706.05350

[90] Zhou B, Khosla A, Lapedriza A, Oliva A, Torralba A. Learning deep features for discriminative localization. In: Proceedings of the IEEE conference on computer vision and pattern recognition. 2016;2921–2929

