# Supplementary material for: Validation of genetic variants from NGS data using deep convolutional neural networks
Source: BMC Bioinformatics. 2023 Apr 20;24:158. doi: 10.1186/s12859-023-05255-7 (PMC10116675; doi:10.1186/s12859-023-05255-7)
Supplement: Supplementary file 1 — Additional file 1. Appendix. [file 12859_2023_5255_MOESM1_ESM.pdf]

Appendix

A Details on bioinformatics pipelines

Tools	In-facility	Out-of-facility	Kotani et al. [74]
BWA-MEM	v0.7.15	v0.7.15	NA
PicardTools	v2.2.2	v2.21.9	NA
GATK	v3.7	v3.7*; v4.1.7.0	NA
samtools	v1.3.1	v1.10	NA
VarScan2	v2.4.2	v2.4.4	NA
ANNOVAR	v2015Dec14	v2017July17	NA

Table S1: List of different versions of bioinformatics tools used for WES data analysis of in-facility training data set, out-of-facility validation data set. Tool versions used by Kotani et al. are not available. NA = not available; *v3.7 used for RealignerTargetCreator, IndelRealigner, DepthOfCoverage.

Variant calling	In-facility	Out-of-facility	Kotani et al. [74]
min-coverage-normal	5	10	10/8
min-coverage-tumor	5	10	10/8
min-var-freq	0.05	0.07	0.07/0.05
somatic p-value	0.05	0.05	0.001 or *0.05/0.003
strand filter	1	no	no

Table S2: List of VarScan2 filter settings used for variant calling of In-facility training data set and Out-of-facility validation data set in house and list of settings used by Kotani et al. *p-value 0.05, if more than 20 percent of the mutated allele in tumour sample and less than 10 percent of the mutated in normal sample

B Examples for library-specific artefacts

Below are two example positions which exhibit consistent sequencing artefacts across a library. Without this context, they could be mistaken for mutations.

Figure S1: **Example 1.** SNV call from comparison of GL_1 (GL111) to CLL_1 (CLL111), but alteration recurs across library preparation, e.g. in a different tumour sample CLL_2 (CLL91), and also an unrelated germline sample GL_2 (GL91). Locus chr9, 55209101

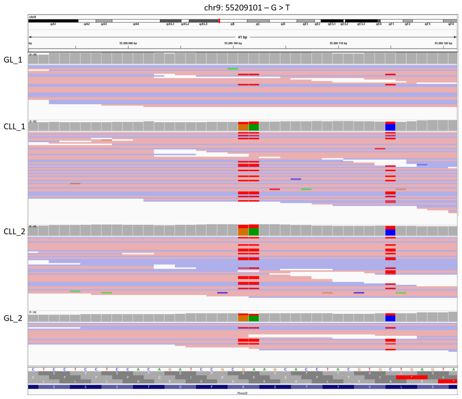

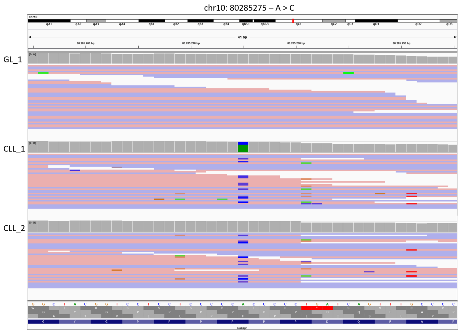

Figure S2: **Example 2.** SNV call from comparison of GL_1 (GL_C25) to CLL_1 (CLL6), but also recurs in the unrelated sample CLL_2 (CLL91). Locus chr10, 80285275

C Cross-validation scores

Below, the individual fold scores for cross-validation with and without context tracks can be found. All values rounded to three decimals.

Fold	Accuracy	Precision	Recall	F1
1	0.954	0.912	0.957	0.934
2	0.974	0.933	0.993	0.962
3	0.966	0.977	0.922	0.949
4	0.974	0.978	0.943	0.96
5	0.974	0.964	0.957	0.961
Average	0.968	0.953	0.954	0.953
Std. dev.	0.008	0.029	0.026	0.012

Table S3: Detailed scores from 5-fold cross-validation with context tracks.

Without context:

Fold	Accuracy	Precision	Recall	F1
1	0.954	0.907	0.965	0.935
2	0.945	0.921	0.915	0.918
3	0.947	0.961	0.879	0.919
4	0.954	0.962	0.9	0.93
5	0.957	0.93	0.943	0.936
Average	0.952	0.936	0.92	0.927
Std. dev.	0.005	0.025	0.034	0.009

Table S4: Detailed scores from 5-fold cross-validation without context tracks.

D Evaluated model architectures

During the process of choosing a suitable model architecture, we experimented with a number of topologies. This included configurations of layers with more powerful classification heads consisting of more dense layers with larger widths, which we however ultimately abandoned to avoid overfitting. In place of the 2D convolutions applied on a per-slice basis to the tensorised data, we briefly considered three-dimensional convolutions, but did not pursue this approach further due to computational limitations.

Moreover, we evaluated a number of techniques considered to provide a regularising effect for convolutional neural networks. These included:

- Direct l^2 regularisation on neuron activity in the dense layers [89],
- augmenting the training data by dropping the ends of individual reads at random with a rate of 0.1,
- scheduling the learning rate of the optimiser to be reduced by a factor of 10 upon encountering an objective function plateau for at least 5 epochs, and
- replacing the convolution-to-dense transition by a Global Average Pooling (GAP) layer [90].

None of these were observed to have a meaningful impact on the model's performance, although an exhaustive grid search of hyperparameters was not carried out for computational reasons.

E Parameters used for rule-based filtering

For the rule-based filtering approach suggested by Wardell et al. [62], we used the following parameter sets (for an explanation of the different criteria, we refer to [62]):

Criterion	Default value	Permissive value
Minimum depth in tumor and normal	10	5
Maximum depth in tumor and normal	1000	1000
Minimum number of ALT reads in tumor	3	2
Minimum median base quality	30	20
Maximum proportion of zero mapping quality reads in tumor and normal	0.05	0.02
Minimum median mapping quality of ALT reads in tumor	50	20
Maximum difference between median mapping quality of ALT reads in tumor and REF reads in normal	5	10
Maximum median shortest distance to either aligned end in tumor	10	15
Minimum MAD of ALT position in tumor	3	2
Maximum edit distance of ALT reads in tumor	4	7
Maximum VAF in normal	0.03	0.15
Minimum VAF in tumor	0.05	0.15
Maximum OAF in tumor	0.04	0.1
Maximum proportion of secondary alignments in tumor	0.05	0.1
Maximum proportion of inversion orientation reads in normal	0.2	0.4
FoxoG artifact proportion	0.9	0.9
